# Supplementary material for: Development of the oral resistome during the first decade of life
Source: Nat Commun. 2023 Mar 9;14:1291. doi: 10.1038/s41467-023-36781-w (PMC9998430; doi:10.1038/s41467-023-36781-w)
Supplement: Supplementary file 1 — Supplementary Information [file 41467_2023_36781_MOESM1_ESM.pdf]

Supplementary Information

Supplementary Tables

Table S1: Differential abundance analysis (MaAsLin2) of the impact of time/stage of dental development on the abundance (TPM) of ARGs. Analysis was performed on ARGs above a minimum prevalence of 0.01%, present in at least 20% of the samples, which had been normalised by total sum scaling (TSS) and log transformed.

ARG	Time point	coef	stderr	N	N.not.0	pval	qval
mef(A)	T3	-0.040	0.009	530	516	1.2E-05	3.8E-05
msr(D)	T3	-0.030	0.006	530	506	3.8E-06	1.6E-05
tetA(46)	T3	-0.026	0.002	530	265	2.7E-31	3.1E-30
tetB(46)	T3	-0.026	0.002	530	264	2.2E-30	2.0E-29
bacA	T3	-0.023	0.006	530	147	7.0E-05	1.8E-04
RlmA(II)	T2	-0.020	0.007	530	513	2.1E-03	4.1E-03
tetA(46)	T2	-0.019	0.002	530	265	2.5E-17	1.9E-16
tetB(46)	T2	-0.019	0.002	530	264	1.1E-16	7.0E-16
patA	T2	-0.016	0.004	530	487	2.5E-05	6.6E-05
patB	T2	-0.011	0.004	530	492	4.1E-03	7.5E-03
mef(E)	T3	-0.007	0.001	530	138	4.8E-06	1.8E-05
tetA(60)	T3	-0.005	0.001	530	260	2.3E-06	1.1E-05
mef(E)	T2	-0.003	0.001	530	138	2.5E-02	4.2E-02
tet(Q)	T3	0.001	0.000	530	191	3.8E-04	8.8E-04
penA	T2	0.011	0.005	530	353	2.9E-02	4.8E-02
APH(6)-Id	T2	0.011	0.003	530	137	3.5E-04	8.5E-04
blaCSP-1	T3	0.011	0.002	530	160	3.6E-08	1.8E-07
sul2	T2	0.012	0.004	530	182	1.6E-03	3.3E-03
penA	T3	0.016	0.005	530	353	8.9E-04	1.9E-03
sul2	T3	0.017	0.004	530	182	1.2E-05	3.8E-05
macB	T2	0.017	0.006	530	439	1.8E-03	3.6E-03
tet(32)	T3	0.019	0.003	530	151	2.7E-11	1.6E-10
blaTEM-1	T2	0.021	0.007	530	242	5.4E-03	9.6E-03
cfxA3	T3	0.030	0.007	530	298	1.4E-05	3.9E-05
cfxA3	T2	0.032	0.007	530	298	6.4E-06	2.3E-05
macB	T3	0.080	0.005	530	439	5.1E-43	7.8E-42
macA	T3	0.090	0.005	530	386	3.4E-59	1.6E-57
lsa(C)	T3	0.102	0.006	530	369	1.6E-44	3.7E-43

Note 1: Reference level is T1, resulting in comparison of T1 vs T2 and T1 vs T3.
Note 2: Negative coefficient values indicate ARG is more abundant at T1 compared to either T2 or T3.
Note 3: Positive coefficient values indicate ARG is more abundant at T2 or T3 compared to T1.

Table S2.1: Relationship between resistome composition (Shannon ARG) and microbiome/species composition (Shannon SP) using a generalised estimating equation.
Age and sex were not adjusted for because neither were found to have a significant effect

Shannon ARG (Outcome)	Shannon SP (Predictor)	N	Coef	se	p-value
Time 1	Time 1	137	0.313	0.049	2.02E-10
Time 2	Time 1	119	-0.076	0.051	0.137
Time 3	Time 1	132	-0.037	0.036	0.299
Time 2	Time 2	178	0.514	0.117	1.08E-05
Time 3	Time 2	165	0.045	0.047	0.336
Time 3	Time 3	206	0.343	0.086	6.61E-05

Table S2.2: Relationship between resistome composition (Shannon ARG) and functional pathways (Shannon Function) using a generalised estimating equation.
Age and sex were not adjusted for because neither were found to have a significant effect.

Shannon ARG (Outcome)	Shannon SP (Predictor)	N	Coef	se	p-value
Time 1	Time 1	137	0.398	0.047	2.91E-17
Time 2	Time 1	119	0.04	0.047	0.393
Time 3	Time 1	132	0.025	0.039	0.513
Time 2	Time 2	178	0.448	0.058	8.49E-15
Time 3	Time 2	165	0.04	0.039	0.297
Time 3	Time 3	206	0.182	0.053	0.001

Table S2.3: Summary statistics for changes over time based on generalised estimating equation analysis.

	T2 – T1 (N = 121)				T3 – T1 (N = 134)				T3 – T2 (N =170)			
	Mean	SD	% Δ	p-value	Mean	SD	% Δ	p-value	Mean	SD	% Δ	p-value
Richness nomdep	5.69	7.56	33.7	1.99E-13	-2.13	6.43	-12.9	0.0002	-8.95	7.27	-39.4	8.76E-36
TPM nomdep*	0.04	0.94	0.3	0.6542	-0.19	1.01	-1.5	0.0274	-0.11	0.82	-0.85	0.0863
Shannon ARG	0.3	0.52	15.1	4.86E-09	0.16	0.51	8.2	0.0004	-0.17	0.51	-7.72	1.38E-05
Shannon SP	0.71	0.66	20.8	7.49E-22	0.96	0.66	28.7	7.95E-35	0.15	0.4	3.61	2.90E-06
Shannon function	0.76	0.62	13.3	1.22E-25	1.16	0.72	20.5	1.04E-38	0.3	0.65	4.61	1.17E-08
Percent resistome	-0.01	0.04	-10	0.0032	-0.05	0.03	-53.5	1.34E-37	-0.04	0.03	-49.3	3.76E-49
IS richness*	0.8	0.71	13.4	3.47E-23	0.8	0.7	13.4	5.36E-26	-0.1	0.59	-1.5	0.0267

*Variable was log transformed.

Table S3.1: Correlation between monozygotic (MZ) and dizygotic (DZ) twins and their 95% confidence intervals (CI) per time point**.** Analysis was conducted using *square and **natural logarithm transformed data.

	N	Corr MZ	95% CI	N	Corr DZ	95% CI
Time point 1						
Shannon_ARG*	28	0.15	(-0.23, 0.50)	33	0.44	(0.16, 0.66)
mefA**	27	0.61	(0.36, 0.78)	29	0.48	(0.15, 0.72)
mrsD**	25	0.55	(0.23, 0.76)	30	0.43	(0.13, 0.66)
penA	13	0.37	(-0.29, 0.79)	10	0.45	(-0.01, 0.74)
RlmA**	24	0.76	(0.57, 0.87)	31	0.48	(0.18, 0.70)
macB**	15	0.2	(-0.43, 0.69)	11	0.41	(-0.04, 0.72)
patB**	23	0.14	(-0.21, 0.46)	25	0.52	(0.17, 0.76)
Time point 2						
Shannon_ARG*	38	0.49	(0.26, 0.66)	51	0.28	(-0.03, 0.54)
mefA**	38	0.66	(0.47, 0.80)	48	0.47	(0.23, 0.65)
msrD**	36	0.68	(0.49, 0.81)	45	0.5	(0.27, 0.68)
penA**	29	0.49	(0.21, 0.69)	37	0.37	(0.05, 0.62)
RlmA**	37	0.63	(0.44, 0.77)	48	0.4	(0.12, 0.62)
macB**	34	0.55	(0.29, 0.73)	46	0.32	(0.05, 0.54)
patB**	35	0.17	(-0.31, 0.58)	47	0.15	(-0.09, 0.37)
Time point 3						
Shannon_ARG*	43	0.52	(0.31, 0.68)	57	0.14	(-0.14, 0.41)
mefA**	42	0.52	(0.30, 0.69)	56	0.33	(0.07, 0.55)
mrsD**	42	0.47	(0.22, 0.66)	56	0.33	(0.07, 0.55)
penA**	19	0.59	(0.27, 0.79)	30	0.44	(0.12, 0.68)
RlmA**	42	0.4	(0.14, 0.61)	55	-0.02	(-0.28, 0.24)
macB**	41	0.33	(0.08, 0.53)	49	0.42	(0.13, 0.65)
patB**	38	0.58	(0.36, 0.74)	51	0.11	(-0.16, 0.37)

Table S3.2: Standardised estimates of genetic effect A (heritability), shared environmental effect C and individual environmental effects E, per time point. Analysis was conducted using *square and **natural logarithm transformed; upper bound of the 95% CI was truncated at 0.99. Significant results are in bold.

	A	95% CI	C	95% CI	E	95% CI
Time point 1						
Shannon_ARG*	0	NA	0.32	**(0.10, 0.55)**	0.67	**(0.45, 0.90)**
mefA**	0.25	(-0.42, 0.92)	0.35	(-0.24, 0.94)	0.39	**(0.18, 0.60)**
msrD**	0.24	(-0.49, 0.96)	0.31	(-0.27, 0.89)	0.45	**(0.19, 0.72)**
penA**	0	NA	0.42	**(0.08, 0.76)**	0.58	**(0.24, 0.92)**
RlmA**	0.56	(-0.01, 0.99)	0.2	(-0.33, 0.72)	0.24	**(0.09, 0.39)**
macB**	0	NA	0.33	(-0.01, 0.67)	0.67	**(0.33, 0.99)**
patB**	0	NA	0.29	**(0.03, 0.55)**	0.71	**(0.45, 0.97)**
Time point 2						
Shannon_ARG*	0.43	(-0.26, 0.99)	0.06	(-0.54, 0.66)	0.51	**(0.31, 0.71)**
mefA**	0.38	(-0.12, 0.88)	0.28	(-0.15, 0.71)	0.34	**(0.17, 0.50)**
msrD**	0.35	(-0.13, 0.84)	0.33	(-0.10, 0.75)	0.32	**(0.16, 0.48)**

penA**	0.23	(-0.50, 0.95)	0.26	(-0.35, 0.87)	0.51	**(0.27, 0.76)**
RlmA**	0.46	(-0.11, 0.99)	0.17	(-0.36, 0.69)	0.37	**(0.20, 0.53)**
macB**	0.46	(-0.18, 0.99)	0.09	(-0.44, 0.62)	0.45	**(0.23, 0.67)**
patB**	0.04	(-0.99, 0.99)	0.13	(-0.54, 0.79)	0.83	**(0.36, 0.99)**
Time point 3						
Shannon_ARG*	0.49	**(0.29, 0.69)**	0	NA	0.51	**(0.31, 0.70)**
mefA**	0.37	(-0.23, 0.97)	0.15	(-0.37, 0.66)	0.48	**(0.29, 0.68)**
msrD**	0.28	(-0.36, 0.91)	0.19	(-0.33, 0.71)	0.53	**(0.32, 0.75)**
penA**	0.31	(-0.43, 0.99)	0.29	(-0.32, 0.88)	0.41	**(0.15, 0.67)**
RlmA**	0.32	**(0.07, 0.57)**	0	NA	0.68	**(0.43, 0.93)**
macB**	0	NA	0.36	**(0.18, 0.54)**	0.64	**(0.46, 0.82)**
patB**	0.53	**(0.31, 0.75)**	0	NA	0.47	**(0.25, 0.69)**

Table S4: Differentially abundant ARGs (TPM) in health, caries and treatment.

ARG		MaAsLin2			ANCOM-BC		
	Value	Co-efficient	q-val	SE	Beta coefficient	q-val	SE
blaOXA-85	mild	-	-	-	-0.30184	0	-0.6956
catP	mild	-	-	-	-0.44425	0	-1.13585
erm(F)	mild	-	-	-	-0.85755	0	-1.29578
tet(B)	mild	-	-	-	-0.00352	0	-0.00593
APH(6)-Id	moderate	-	-	-	-0.57338	0	0.70145
blaOXA-85	moderate	-	-	-	-0.13334	0	0.519489
catP	moderate	-	-	-	1.123747	0	0.849281
cfxA3	moderate	-	-	-	0.097652	0	0.340498
msr(D)	moderate	-	-	-	1.08474	0.018932	0.355398
patA	moderate	-	-	-	1.318942	0.013736	0.386476
penA	moderate	-	-	-	1.421554	0.013736	0.435539
sul2	moderate	-	-	-	1.047821	0.024851	0.363821
tet(32)	moderate	-	-	-	-2.14077	0	0.906475
tet(Q)	moderate	-	-	-	0.630346	0	0.731191
tetA(60)	moderate	-	-	-	-0.88257	0	0.645691
tetB(60)	moderate	-	-	-	-0.63833	0	0.745744
APH(6)-Id	severe	-	-	-	-0.3864	0	0.951781
blaOXA-85	severe	-	-	-	0.073479	0	0.660692
catP	severe	-	-	-	-0.21373	0	0.493743
erm(F)	severe	-	-	-	-0.28466	0	0.917407
tet(B)	severe	-	-	-	0.517717	0	0.777672
tet(W)	severe	-	-	-	-1.19175	0	0.666139
tetA(60)	severe	-	-	-	-1.25816	0	0.729116
blaOXA-85	restored_yes	-	-	-	-0.46342	0	-1.0285
tet(Q)	restored_yes	-	-	-	-0.53652	0	-0.73221

Note 1: No significant results were found using MaAsLin2
Note 2: Reference (Value) is Healthy
Note 3: Negative coefficient values indicate ARG is more abundant in health
Note 4: Positive coefficient values indicate ARG is more abundant in caries

Table S5. Demographic and lifestyle characteristics of the study cohort (n=221).
NA = no information available.

	Characteristic	Sample
Sex	Female	124
	Male	97
Zygosity	Monozygous (MZ)	93
	Dizygous (DZ)	128
Ethnicity*	White	207
	Asian	6
	Mixed	8
Socio-economic status**	High	130
	Low	91
Mode of delivery	Vaginal	45
	Caesarean section	176
Exclusively breast fed for 6 months	Yes	36
	No	38
	Breast and formula	143
	NA	4
Caries status at T3 (n =211)	ICDAS II Score 0	145
	ICDAS II Score 1	14
	ICDAS II Score 2	14
	ICDAS II Score 3	13
	ICDAS II Score 4	6
	ICDAS II Score 5	11
	ICDAS II Score 6	8
Antibiotic Exposure	T1 (n = 139)	118
	T2 (n = 180)	104
	T3 (n = 211)	90
Diet*** at T3	Below	77
	Meets	21
	Above	34
	NA	79

Ethnicity* was defined using the NIH Racial and Ethnic Categories
Socio-economic status** was defined by postcode. SES status was generated by referencing the postcodes against the Index of Relative Socio-economic Advantage and Disadvantage (IRSAD) scores created by the Australian Bureau of Statistics IRSAD score scale of 1-5 was collapsed into two categories – low (1-2) and high (3-5).
Diet*** at T3 focuses on recommended meat, poultry and fish servings for Australian children age and sex matched for the cohort.

Supplementary Figures

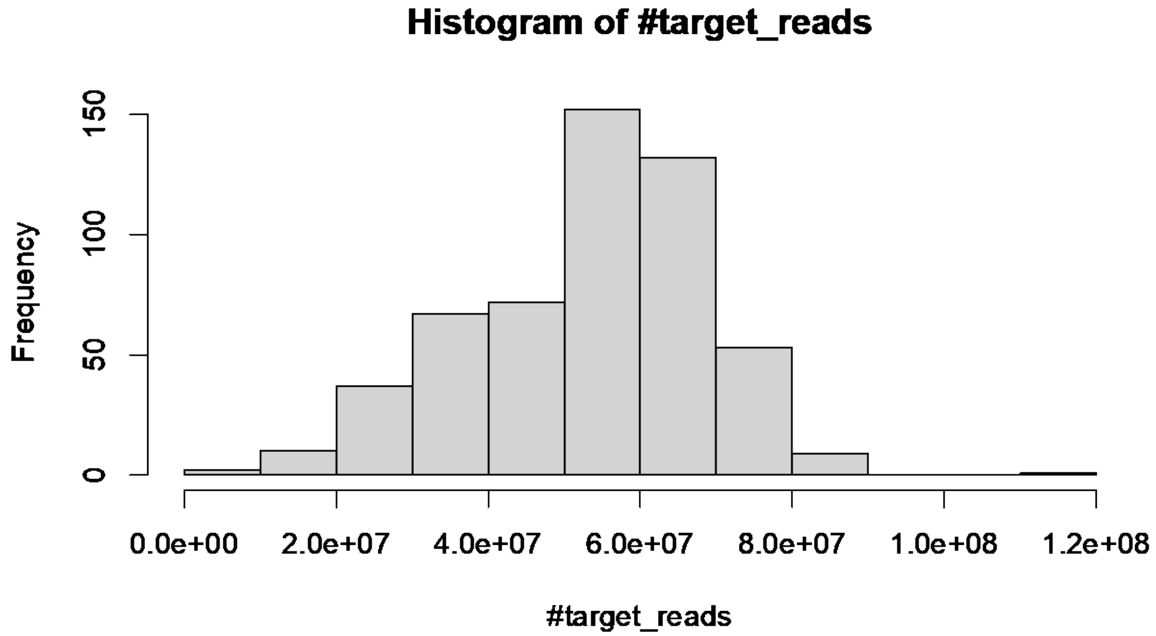

Figure S1: Histogram of target reads sequence depth. Sequences were filtered to remove adapters and host/human mapped sequences. Histogram displays the frequency or number of samples by read depth, revealing the majority of samples fell within less than an order of magnitude range.

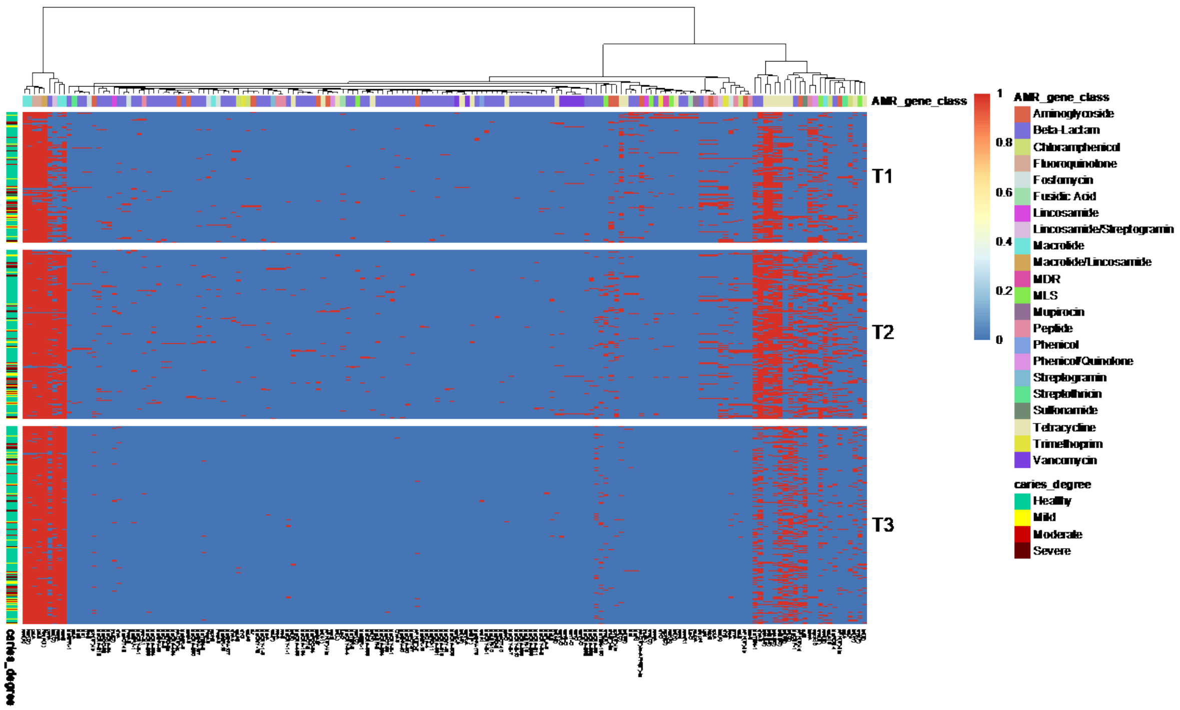

Figure S2. Heatmap showing distribution of antimicrobial resistance genes across all samples by time point. ARGs were treated as binary, present = red and absent = blue. Order of ARGs was determined by hierarchical clustering using wards linkage and coloured by AMR gene class.

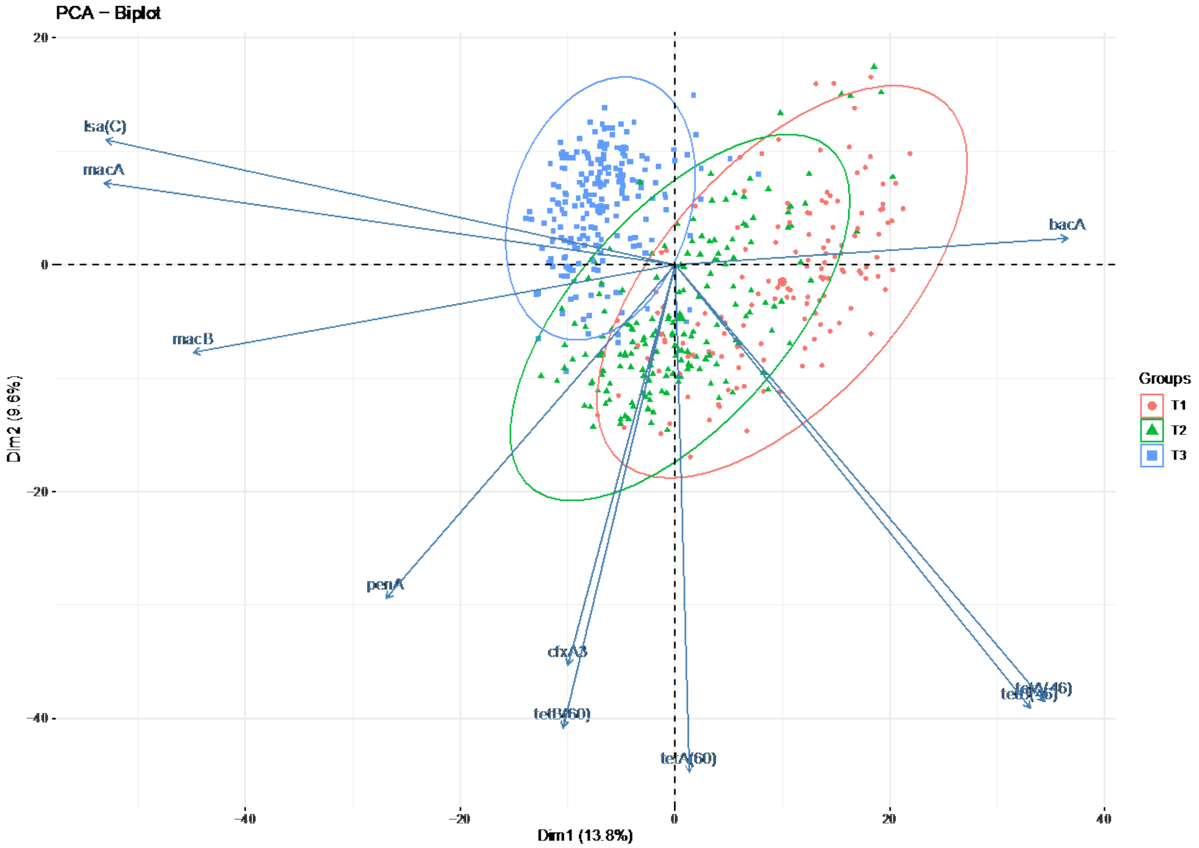

Figure S3. Principal Component Analysis Biplot. Analysis was performed on ARGs above a minimum prevalence of 0.01% that had been normalised by total sum scaling (TSS) and center log ratio (CLR) transformed. A biplot was created by displaying the ARGs which had the top ten vector loading values.

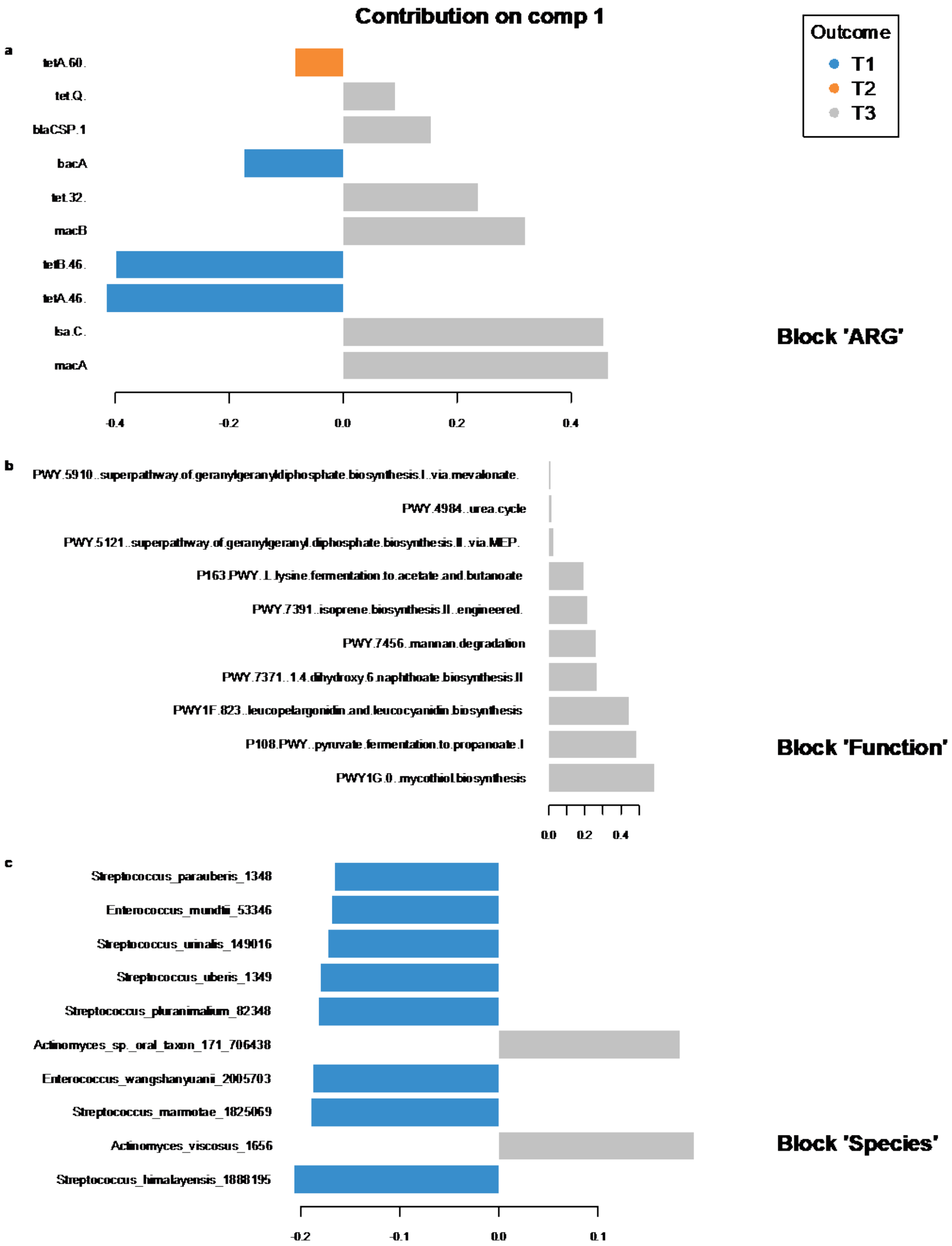

Figure S4. Vector loadings for ARGs, functional pathways and species that contribute to discrimination of individuals by time point/stage of dental development from DIABLO analysis. For each data type, DIABLO was performed on features above 0.01% abundance that had been TSS and CLR transformed. For each data block, displayed are the top ten features (ARG, function and species) with the largest vector loadings.

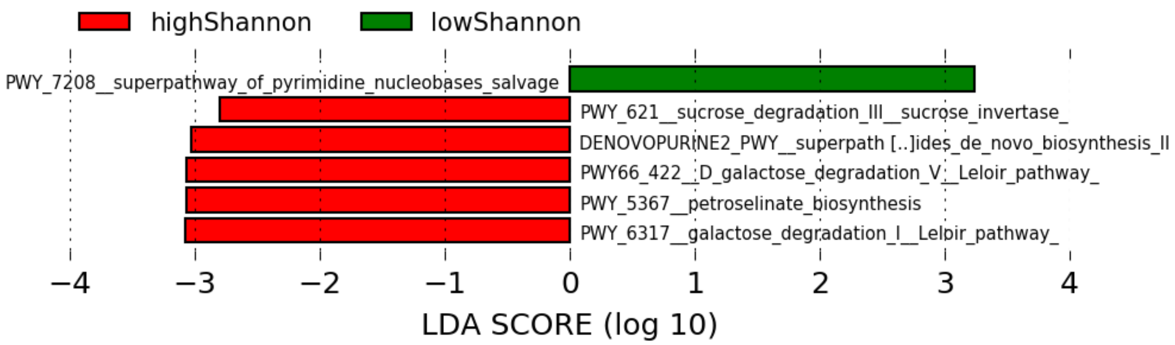

Figure S5: LEfse analysis of 66 biofilm related functional pathways that are discriminatory between individuals with high and low resistome diversity at T3. Lefse was performed on Counts Per Million normalised, biofilm-related pathways. Diversity grouping was defined by the distribution of Shannon Index calculated from the abundance of ARGs (TPM normalised). Individuals were categorised as high diversity if Shannon score >2.10, or low diversity if Shannon score <= 2.10.

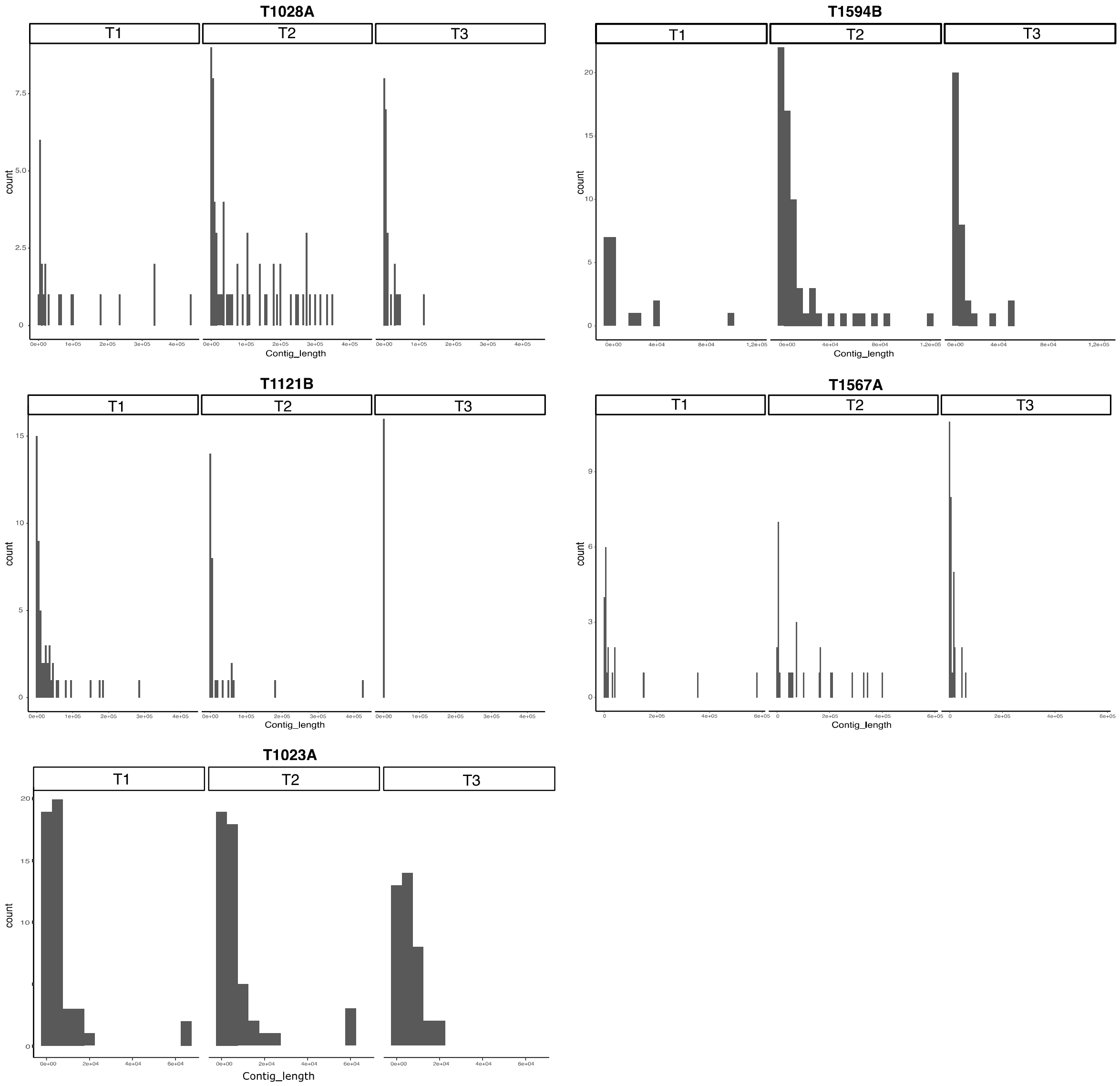

Figure S6. Distribution of lengths of ARG carrying contigs. The distribution length of contigs carrying ARGs was assessed from five randomly selected samples (T1028A, T1594B, T1121B, T1667A and T1023A) at T1 (7.2 mths), T2 (1.6 yrs) and T3 (8.8 yrs). Bin width was set at 5000.

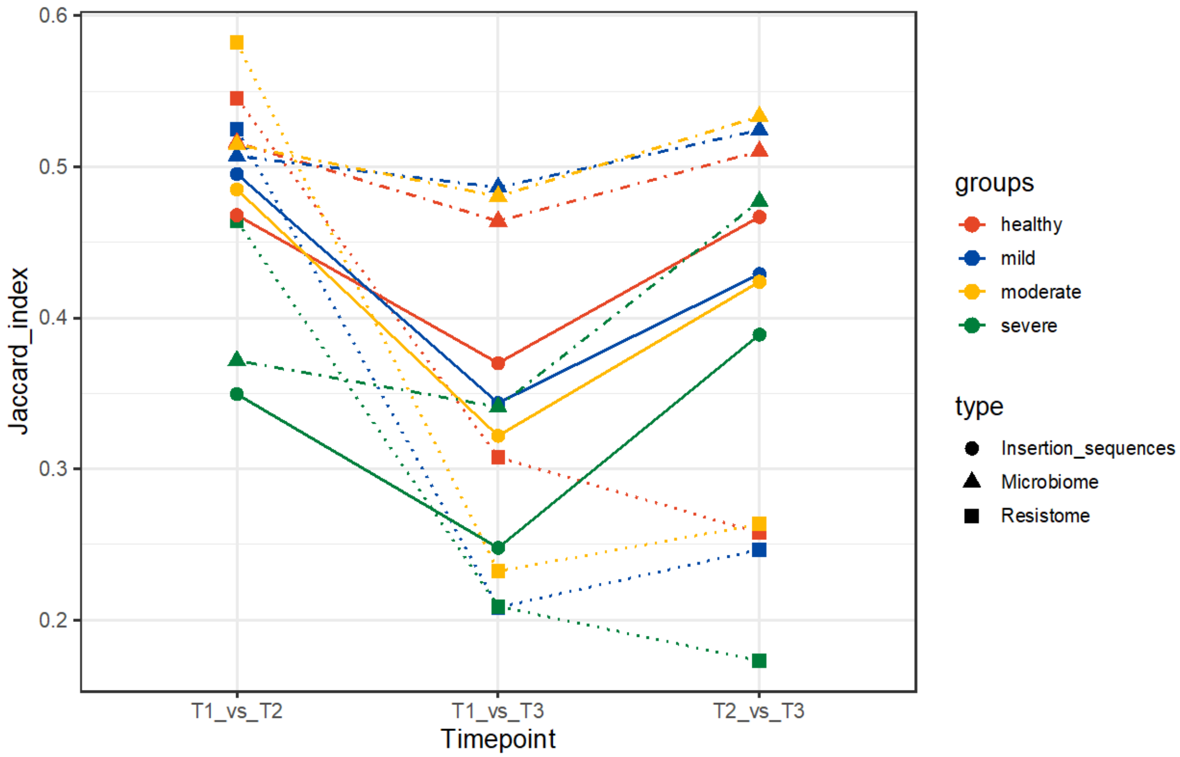

Figure S7. Jaccard index of the richness of species (microbiome), insertion sequences (IS) and ARGs (resistome), between each two time points based on oral health status. Richness calculations were performed on the abundance of species (counts per million normalised), IS (transcripts per million normalised) and ARGs (transcripts per million normalised). Samples were categorised into 4 oral health groups based on the whole mouth ICDAS score: 0: healthy; 1-2: mild caries; 3-4: moderate caries; 5-6: severe caries.

THE UNIVERSITY OF ADELAIDE AUSTRALIA

School of Dentistry

Tooth Emergence and Oral Health in Twins and Their Families

The Easy Steps to Collecting Your Family's Plaque Samples

Materials provided:

(a) 2 packs of sterilized swabs marked Sample 1 and Sample 2. Each pack contains 1 swab per family member;

(b) 2 packs of tubes marked Sample 1 and Sample 2. Each pack contains 1 tube per family member; with the family member's name highlighted on the label;

(c) reply-paid addressed padded bag for the return of all tubes to The University of Adelaide.

When to collect the plaque samples

For best results we would like you to collect the plaque samples twice on the same day, **morning and afternoon/evening**, prior to your regular brushing time, and at least an hour after food or drink. If same-day sampling is not possible, Sample 2 on the next day is ok. **Choosing a day when you will be able to post the samples soon afterwards is ideal.**

How to collect plaque (which is invisible to the naked eye)

Sample 1 –
We would like you to ***gently*** swab around the mouth including the gums, teeth and tongue for approximately 5-10 seconds with a swab from Sample 1 pack. Any attached saliva (dribble) is desirable! Holding the stick in the palm of your hand, with your index finger about 2 cm from the swab will mean your baby will be more likely to grab your hand than the stick!

After swabbing **is completed**, pick up corresponding tube with the correct name, open lid, insert cotton end of swab into tube and snap off as per diagram (two hands needed!). The agar solution (seaweed jelly, sugar, preservatives) is in solid form and stays inside the tube as you insert the swab. Firmly close the lid of the tube and place in return pack. Safely discard the remaining stick of the swab.

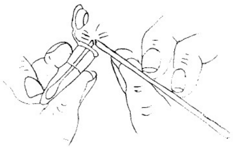

Sample 2
Repeat the above procedure with **new swabs** provided for **Sample 2**.

After completion, please post padded bag at your earliest convenience – no stamps required!

Thank you for collecting plaque samples for the study

"Tooth emergence and oral health in twins and their families".

Note: swabs may appear discoloured, a result of the sterilisation process – swabs are clean!

Figure S8. Instruction pamphlet provided to parents/care givers regarding collection of oral biofilm samples at time points 1 and 2**.**

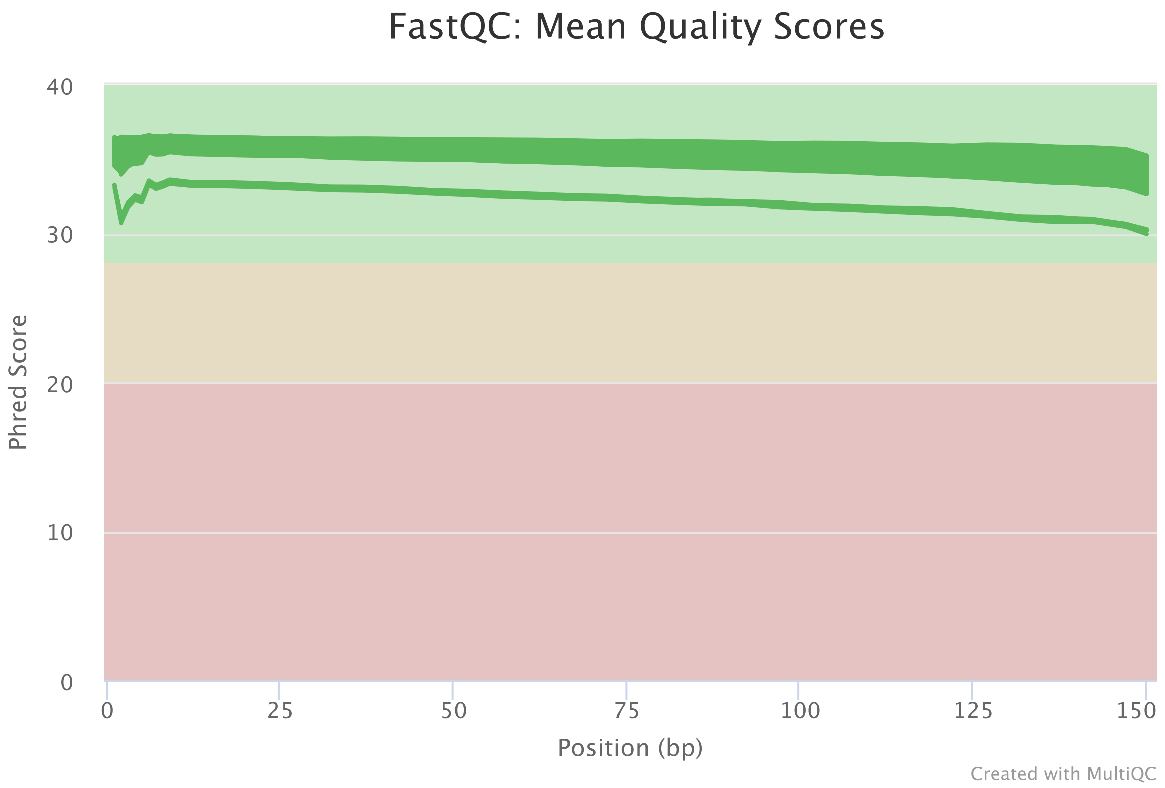

Figure S9. Mean quality scores and per-sequence quality scores for raw fastq sequencing data. Quality scores were assessed with FastQC and aggregated with MultiQC. Data shown is for all sequenced samples, including those later filtered as replicates, low quality or containing high host (> 65%) contamination.
