# Supplementary material for: Development of the oral resistome during the first decade of life
Source: Nat Commun. 2023 Mar 9;14:1291. doi: 10.1038/s41467-023-36781-w (PMC9998430; doi:10.1038/s41467-023-36781-w)
Supplement: Supplementary file 3 — Description of Additional Supplementary Files [file 41467_2023_36781_MOESM3_ESM.docx]

# **Supplementary Data Legends**

File Name: Supplementary Dataset 1

Description: Sequence metrics for all 530 samples.

File Name: Supplementary Dataset 2

Description: Antimicrobial resistance genes (ARGs) identified in our cohort.

File Name: Supplementary Dataset 3

Description: Correlation analysis (DIABLO) between predicted functional pathways, ARGs and species.

File Name: Supplementary Dataset 4

Description: Species names and reference sequences used to generate 16S phylogeny.

File Name: Supplementary Dataset 5

Description: List of species associated with ARG carrying contigs.

File Name: Supplementary Dataset 6

Description: List of insertion sequences identified at all time points.

File Name: Supplementary Dataset 7

Description: Contigs carrying ARGs and *Tn916* within 30kb.

File Name: Supplementary Dataset 8

Description: Complete metaSupplementary Dataset for the cohort at all time points.

File Name: Supplementary Dataset 9

Description: Analysis of association between environmental factors and resistome composition using linear mixed models.

File Name: Supplementary Dataset 10

Description: Relative abundance of all ARGs (Transcripts per million [TPM]) at T1.

File Name: Supplementary Dataset 11

Description: Relative abundance of all ARGs (TPM) at T2.

File Name: Supplementary Dataset 12

Description: Relative abundance of all ARGs (TPM) at T3.
