# Supplementary material for: Development of the oral resistome during the first decade of life
Source: Nat Commun. 2023 Mar 9;14:1291. doi: 10.1038/s41467-023-36781-w (PMC9998430; doi:10.1038/s41467-023-36781-w)
Supplement: Supplementary file 16 — Reporting Summary [file 41467_2023_36781_MOESM16_ESM.pdf]

nature portfolio

Corresponding author(s): Smitha Sukumar and Christina Adler

Last updated by author(s): Feb 5, 2023

Reporting Summary

Nature Portfolio wishes to improve the reproducibility of the work that we publish. This form provides structure for consistency and transparency in reporting. For further information on Nature Portfolio policies, see our Editorial Policies and the Editorial Policy Checklist.

Statistics

For all statistical analyses, confirm that the following items are present in the figure legend, table legend, main text, or Methods section.

n/a	Confirmed	
☐	☒	The exact sample size (*n*) for each experimental group/condition, given as a discrete number and unit of measurement
☐	☒	A statement on whether measurements were taken from distinct samples or whether the same sample was measured repeatedly
☐	☒	The statistical test(s) used AND whether they are one- or two-sided<br>*Only common tests should be described solely by name; describe more complex techniques in the Methods section.*
☐	☒	A description of all covariates tested
☐	☒	A description of any assumptions or corrections, such as tests of normality and adjustment for multiple comparisons
☐	☒	A full description of the statistical parameters including central tendency (e.g. means) or other basic estimates (e.g. regression coefficient) AND variation (e.g. standard deviation) or associated estimates of uncertainty (e.g. confidence intervals)
☐	☒	For null hypothesis testing, the test statistic (e.g. *F*, *t*, *r*) with confidence intervals, effect sizes, degrees of freedom and *P* value noted<br>*Give P values as exact values whenever suitable.*
☒	☐	For Bayesian analysis, information on the choice of priors and Markov chain Monte Carlo settings
☒	☐	For hierarchical and complex designs, identification of the appropriate level for tests and full reporting of outcomes
☒	☐	Estimates of effect sizes (e.g. Cohen's *d*, Pearson's *r*), indicating how they were calculated

Our web collection on statistics for biologists contains articles on many of the points above.

Software and code

Policy information about availability of computer code

Data collection	No software was used for data collection.

Data analysis

Details of our bioinformatics pipeline which uses open source software: Willet C, Martinez, E., Sukumar, S., Adler, C., Lydecker, H., Wang, F., Chew, T., & Sadsad, R. https://github.com/Sydney-Informatics-Hub/Shotgun-Metagenomics-Analysis. 1.0 edn. This pipeline uses the following software, FastQC v0.11.7, MultiQC v1.7 , BBtools 'BBmap' v37.98, MEGAHIT v1.2.8 , SAMtools v1.10, ABRicate v0.9.9, Kraken2 v. 2.08, Bracken v2.6.0, Prokka v. 1.14.5, HUMAnN2 v2.8.2 and Prodigal v2.6.3.
MaAslin2 v1.0.0 (Differential Abundance analysis): Lin H, Peddada SD. Analysis of compositions of microbiomes with bias correction. Nat Commun 11, 3514 (2020).
ANCOM-BC v2.1.2 (Differential abundance analysis): Mallick H, et al. Multivariable association discovery in population-scale meta-omics studies. Plos Comp Biol 17, 11 (2021).
DIABLO v6.10.9 (multi-correlation analysis): Singh A, Shannon CP, Gautier B, Rohart F, Vacher M, Tebbutt SJ, Lê Cao KA. DIABLO: an integrative approach for identifying key molecular drivers from multi-omics assays. Bioinformatics. 2019 Sep 1;35(17):3055-3062.
LEfSe v1.0.0 Linear discriminant analysis Effect Size): Segata, N., Izard, J., Waldron, L. et al. Metagenomic biomarker discovery and explanation. Genome Biol 12, R60 (2011). https://doi.org/10.1186/gb-2011-12-6-r60.
Classic Twin modeling: Neale M, Cardon LR. Methodology for genetic studies of twins and families. Springer Science & Business Media (2013).
Vegan package in R: Jari Oksanen FGB, Michael Friendly, Roeland Kindt,, Pierre Legendre DM, Peter R. Minchin, R. B. O'Hara,, Gavin L. Simpson PS, M. Henry H. tevens, Eduard Szoecs,, Wagner H. Community Ecology Package.). 2.5-7 edn (2020).
Mets package in R: Scheike TH, Holst KK, Hjelmborg JB. Estimating heritability for cause specific mortality based on twin studies. Lifetime Data Anal 20, 210-233 (2014).
Team RC. R: A language and environment for statistical computing. (2013).

For manuscripts utilizing custom algorithms or software that are central to the research but not yet described in published literature, software must be made available to editors and reviewers. We strongly encourage code deposition in a community repository (e.g. GitHub). See the Nature Portfolio guidelines for submitting code & software for further information.

Data

Policy information about availability of data

All manuscripts must include a data availability statement. This statement should provide the following information, where applicable:

- Accession codes, unique identifiers, or web links for publicly available datasets
- A description of any restrictions on data availability
- For clinical datasets or third party data, please ensure that the statement adheres to our policy

Sequence data that support the findings of this study has been deposited in European Nucleotide Archive with the project accession code PRJEB54673 (https://www.ebi.ac.uk/ena/browser/view/PRJEB54673). The authors declare that all other data supporting the findings of this study (including source data for all figures) are available within the article, provided in the supplementary information files. Databases used to analyse sequence data included: NCBI AMRFinder Plus (https://www.ncbi.nlm.nih.gov/pathogens/antimicrobial-resistance/AMRFinder/), Resfinder (https://cge.cbs.dtu.dk/services/ResFinder/), Comprehensive Antibiotic Resistance Database (CARD) (https://card.mcmaster.ca/), Kraken 'standard' database (https://ccb.jhu.edu/software/kraken/) and ISFinder database (https://isfinder.biotoul.fr/).

Human research participants

Policy information about studies involving human research participants and Sex and Gender in Research.

Reporting on sex and gender

The findings apply to both sexes. Sex, not gender, was considered in the study design and determined by self (parental) reporting. Details of numbers per sex are included in the paper, with 124 females and 97 males included in the study. Sex-specific analyses were conducted to assess the influence of sex on outcomes.

Population characteristics

The covariate population data is recorded in Supplementary Table S14. In brief, the study included 93 monozygotic and 128 dizygotic twins, that were sampled at three time points, across the ages of 6.7 ± 2.7 months to 8.5 ± 1.2 years old. This included 207 individuals of who categorised themselves as 'White', 6 as 'Asian' and 8 as 'Mixed'. Population characteristics reported included the mode of delivery (45 = vaginally and 176 = cesarean delivered), early feeding (36 = breast, 38 = bottle or 143 = combination feeding), caries status at T3(ICDAS codes, 0 = 145, 1 = 14, 2 = 14, 3 = 13, 4 = 6, 5 = 11 and 6 = 8), antibiotic exposure at T3 (Yes = 90, No = 121) and protein standard at T3 (either Meeting standard = 21, Below = 77 or Above = 34).

Recruitment

A national sample of twins born between 2004-2006 and their families were recruited using the national Australian Twin Registry. In the original study, 1172 individuals were enrolled, with roughly a third being monozygotic twins. From this larger cohort we selected 221 individuals enrolled in the study who were able to attend oral health clinical assessments, that represent approximately equal numbers of both males and females, and monozygotic and dizygotic pairs. The cohort is predominantly of European ancestry, which is to be expected given the demographics of the country when recruitment commenced in 2004. Invitation to attend twin clinics and be part of the current study was open to all study participants. The main selection bias was for families who were interested in oral health and hence potentially had children with better oral health than the general population. This self-selection bias to take part in the study, may have meant we had an under-representation of children with dental caries compared to the general population. Hence, our study is more likely to show subtle as opposed to larger differences between the oral health states.

Ethics oversight

University of Adelaide, Human Ethics Research Committee (H-2013-097 and H-78-2003)

Note that full information on the approval of the study protocol must also be provided in the manuscript.

Field-specific reporting

Please select the one below that is the best fit for your research. If you are not sure, read the appropriate sections before making your selection.

- [x] Life sciences
- [ ] Behavioural & social sciences
- [ ] Ecological, evolutionary & environmental sciences

For a reference copy of the document with all sections, see nature.com/documents/nr-reporting-summary-flat.pdf

Life sciences study design

All studies must disclose on these points even when the disclosure is negative.

Sample size	Sample population was designed to provide a meaningful statistical results, assessing 221 in the present submission with 55% of the children sampled at all three time points. A sample size calculation was not performed. The rationale for deciding on the sample size was determined by assessing current literature, and using either the same or a greater sample number than comparable studies on the oral microbiome, using metagenomic methods.
Data exclusions	There was only one pre-established data exclusion criteria - recent exposure to antibiotics i.e currently taking antibiotics or taken antibiotics in the past three months. Two children (at T3) were excluded from the study based on this criteria. Post-sequencing, we excluded 10 samples due to either sequence depth issues (n = 3) or high contamination with human DNA (n = 7).
Replication	Each sample was a combined sample from two duplicate samples. Each duplicate sample was extracted independently from each other, on different days. The duplicate DNA extracts were then combined for DNA sequencing. Reproducibility of sequencing findings was assessed by sequencing 12 replicates. Reproducibility of analysis, such as differential abundance analysis, was run multiple times and using different platforms.
Randomization	Randomisation of samples was performed before DNA extraction and sequencing.
Blinding	Laboratory researchers performing the DNA extraction and sequencing were blind to the clinical (caries) outcome.

Reporting for specific materials, systems and methods

We require information from authors about some types of materials, experimental systems and methods used in many studies. Here, indicate whether each material, system or method listed is relevant to your study. If you are not sure if a list item applies to your research, read the appropriate section before selecting a response.

Materials & experimental systems

n/a	Involved in the study	
☒	☐	Antibodies
☒	☐	Eukaryotic cell lines
☒	☐	Palaeontology and archaeology
☒	☐	Animals and other organisms
☒	☐	Clinical data
☒	☐	Dual use research of concern

Methods

n/a	Involved in the study	
☒	☐	ChIP-seq
☒	☐	Flow cytometry
☒	☐	MRI-based neuroimaging
